# Structure and Function of the Small MutS-Related Domain

**DOI:** 10.4061/2011/691735

**Published:** 2011-07-19

**Authors:** Kenji Fukui, Seiki Kuramitsu

**Affiliations:** ^1^RIKEN SPring-8 Center, Harima Institute, 1-1-1, Kouto, Sayo-cho, Sayo-gun, Hyogo 679-5148, Japan; ^2^Department of Biological Sciences, Graduate School of Science, Osaka University, 1-1, Machikaneyama-cho, Toyonaka, Osaka 560-0043, Japan

## Abstract

MutS family proteins are widely distributed in almost all organisms from bacteria to human and play central roles in various DNA transactions such as DNA mismatch repair and recombinational events. The small MutS-related (Smr) domain was originally found in the C-terminal domain of an antirecombination protein, MutS2, a member of the MutS family. MutS2 is thought to suppress homologous recombination by endonucleolytic resolution of early intermediates in the process. The endonuclease activity of MutS2 is derived from the Smr domain. Interestingly, sequences homologous to the Smr domain are abundant in a variety of proteins other than MutS2 and can be classified into 3 subfamilies. Recently, the tertiary structures and endonuclease activities of all 3 Smr subfamilies were reported. In this paper, we review the biochemical characteristics and structures of the Smr domains as well as cellular functions of the Smr-containing proteins.

## 1. Introduction

MutS is a key enzyme in DNA mismatch repair (MMR) that corrects mismatched bases produced during DNA replication and other biological processes [1, 2]. MutS recognizes mismatches and stimulates the downstream reactions in MMR [3–5]. MutS orthologues are conserved in almost all organisms including viruses, archaea, bacteria, and eukaryotes [6]. Accumulating genome information has revealed that proteins partly homologous to MutS also exist in all 3 domains of life [7–9]. Among those MutS paralogues, bacterial MutS2 has been relatively well characterized [10, 11]. Although disruption of *mutS2* in* Bacillus subtilis* and *Deinococcus radiodurans *did not affect the phenotype of either strain [12, 13], it was reported that knockout of *mutS2* in *Helicobacter pylori *caused an increase in the frequency of homologous recombination [10, 11]. This result suggests an inhibitory role for MutS2 in homologous recombination. Biochemical characterization demonstrated that, in *Helicobacter pylori *and* Thermus thermophilus*, MutS2 preferably binds to branched DNA structures, including Holliday junction and D-loop structure [10, 11, 14], the intermediates in homologous recombination. Finally, it was confirmed that* T. thermophilus* MutS2 endonucleolytically digests those branched DNA structures [14], indicating that MutS2 suppresses homologous recombination through the resolution of early intermediates in the process. 

The endonuclease domain is located in the C-terminal region of MutS2, which is called the small MutS-related (Smr) domain [15]. While the Smr domain is not found in other MutS homologues, as initially pointed out by Moreira and Philippe [15], sequences homologous to the Smr domain are ubiquitous among a variety of proteins and are conserved in almost all organisms except in archaea (Table 1) [11, 16, 17]. Malik and Henikoff [17] predicted that the Smr domain has an endonuclease activity based on the domain-architecture analogy of MutS2 with *Sgmt-*MutS, a MutS homologue present in *Sarcophyton glaucum* mitochondria. *Sgmt-*MutS contains an HNH endonuclease domain in its C-terminal region [9]. Although the Smr domain has no sequence similarity with the HNH endonuclease domain, the endonuclease activity of Smr domain has been verified by an accumulating amount of experimental evidences [18–20]. In this paper, we review the recent reports about the functional and structural characterization of the Smr domains and discuss the cellular functions of Smr-containing proteins. 

## 2. Smr Domains Can Be Categorized into 3 Subfamilies

**Figure 1:**
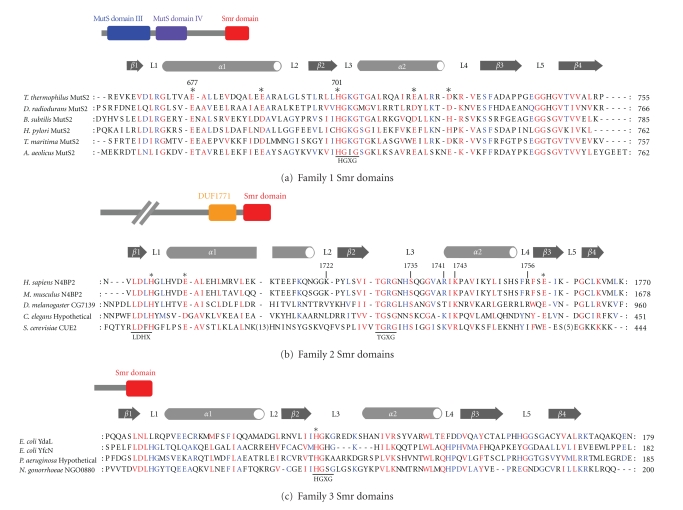
Amino acid sequence alignments of family 1, 2, and 3 Smr domains. (a) Family 1 Smr domains. The schematic representation of* T. thermophilus* MutS2 is shown at the top of the panel. The location of the secondary structure elements of *T. thermophilus* MutS2 Smr domain is shown above the sequences. Glu-677 and His-701 in *T. thermophilus* MutS2 are indicated by numbers above the sequences. *Red* and *blue* characters indicate residues whose chemical characteristics are conserved in all and 5 of the 6 species, respectively. The highly conserved HGKG motif is underlined. The 100% conserved acidic residues are indicated with asterisks. (b) Family 2 Smr domains. The schematic representation of *H. sapiens *N4BP2 is shown at the top of the panel. The location of the secondary structure elements of the Smr domain of *H. sapiens* N4BP2 is shown above the sequences. Lys-1722, Ser-1735, Arg-1741, Lys-1743, and Arg-1756 are indicated by numbers above the sequences. *Red* and *blue* characters indicate residues whose chemical characteristics are conserved in all and 4 of the 5 species, respectively. The 100% conserved acidic residues are indicated with asterisks. (c) Family 3 Smr domains. The schematic representation of *E. coli *YdaL is shown at the top of the panel. The location of the secondary structure elements of* E. coli* YdaL is shown above the sequences. *Red* and *blue* backgrounds indicate residues whose chemical characteristics are conserved in all and 4 of the 5 species, respectively. Perfectly conserved basic residues are indicated with asterisks. The 100% conserved acidic residues are indicated by asterisks.

Smr domains can be categorized roughly into 3 subfamilies on the basis of the domain architecture of the proteins in which the Smr domains are included (Figure 1). The first of these is the C-terminal domain of the MutS2 protein that is found in *Firmicutes*, *Deinococcus-Thermus*, *Bacteroidetes*, *Deltaproteobacteria*, and *Epsilonproteobacteria* phyla of bacteria and plants. Plant MutS2 is believed to be derived from the genomes of incorporated cyanobacteria [7]. In this paper, we refer to this type of Smr domain as a family 1 Smr domain (Figure 1(a)). The second subfamily consists of the C-terminal domains of proteins other than MutS2. For example, the C-terminal domain of the human NEDD4-binding protein 2 (N4BP2) has a significant amino acid sequence homology with the Smr domain of MutS2 [21]. We refer to this type as family 2 Smr domains (Figure 1(b)). The family 2 Smr domains are usually found in eukaryotes. The last group consist of the stand-alone type Smr domains, such as *E. coli *YdaL and *E. coli* YfcN. Proteins belonging to this subfamily exist in both prokaryotes and eukaryotes. In general, MutS2 and the stand-alone type Smr domains do not coexist within the same organism, except for a few species [8, 11]. We refer to the stand-alone type Smr domain as family 3 Smr domains (Figure 1(c)). The amino acid sequence alignments revealed some differences between these 3 Smr subfamilies (Figures 1(a), 1(b), and 1(c)). The HGXG motif (underlined in Figures 1(a) and 1(c)) is characteristic for family 1 and 3 Smr domains. On the other hand, family 2 Smr domains contain a TGXG motif (underlined in Figure 1(b)) rather than the HGXG motif and a highly conserved LDXH motif in their N-terminal regions (underlined in Figure 1(b)). It should be mentioned that motifs somewhat similar to the LDXH are also found in family 1 and 3 Smr domains, implying the significance of this region in the function of Smr domains.

## 3. Structures of the 3 Smr Subfamilies

**Figure 2:**
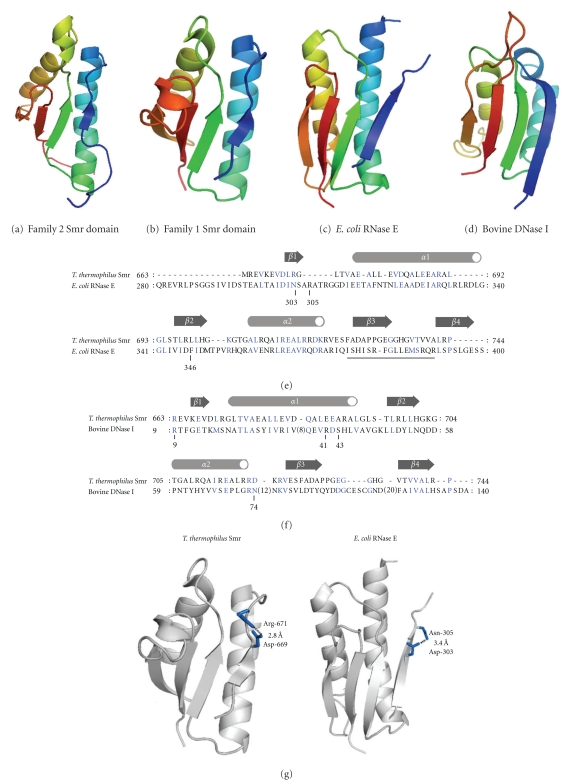
Three-dimensional structures of Smr domains. (a) Solution structure of human N4BP2 Smr domain, a family 2 Smr domain (2d9i). (b) Crystal structure of* T. thermophilus *MutS2 Smr domain, a family 1 Smr domain (2zqe). (c) Crystal structure of the catalytic domain of *E. coli *RNase E (2c0b) [22]. (d) Crystal structure of the N-terminal domain of bovine DNase I (1dnk) [23]. (e) Amino acid sequence comparison between *T. thermophilus* MutS2 Smr domain and the catalytic domain of *E. coli *RNase E. The location of the secondary structure elements of the Smr domain is shown above the sequence. The residues essential for the catalytic activity of RNase E are shown by numbers below the sequence. The dimeric interface in the *E. coli *RNase E catalytic domain is underlined. (f) Amino acid sequence comparison between *T. thermophilus* MutS2 Smr domain and the N-terminal domain of bovine DNase I. The location of the secondary structure elements of the Smr domain is shown above the sequence. The residues essential for the DNA-binding ability are shown by numbers below the sequence. (g) *Left*: a salt bridge between Asp-669 and Arg-671 in the *T. thermophilus *Smr domain is represented with a broken line. *Right*: A hydrogen bond between Asp-303 and Asn-305 is also represented with a broken line.

The solution structure of a family 2 Smr domain, namely, the C-terminal domain of human N4BP2 has been solved (PDB ID: 2D9I (unpublished), and 2VKC [19]). The overall structure of the human N4BP2 C-terminal domain comprises an *α*/*β*-sandwich structure with a *βαβαββ* fold consisting of a four-stranded *β*-sheet stacked against 2 *α*-helices (Figure 2(a)). As shown in Figure 2(b), the crystal structure of a family 1 Smr domain, the* T. thermophilus* MutS2 C-terminal domain (2ZQE), has also been determined, and it shows the same overall structure as the human N4BP C-terminal domain (Z-score: 10.65; root mean square deviation (r.m.s.d.): 2.2 Å; sequence identity: 27%) [14]. Although the coordinate file has not yet been released, the crystal structure of a family 3 Smr domain, residues 39–175 of *E. coli* YdaL, was reported [20]. The overall structure of residues 86–170 of* E. coli* YdaL is also similar to those of the C-terminal domains of human N4BP2 and *T. thermophilus* MutS2. Thus, all 3 Smr subfamilies share a common overall structure as expected from their sequence similarity. As pointed out by Gui et al. [20], the largest structural difference among the 3 Smr subfamilies was found in the length of Loop3 that contains the highly conserved HGXG/TGXG motif. The family 2 Smr domain has the longest loop, while the family 1 Smr domain has the shortest one. 

 Structural homology search program suggested similarity between Smr domains and a variety of proteins including nucleotide-binding proteins [19]. Interestingly, the overall structure of* T. thermophilus* MutS2 Smr domains shows homology to those of the catalytic domain of* E. coli *RNase E (2BX2) (Z-score: 6.63; r.m.s.d: 2.9 Å; sequence identity: 11%) and the N-terminal DNA-binding domain of bovine DNase I (2DNJ) (Z-score: 3.4; r.m.s.d.: 3.0 Å; sequence identity: 10%) (Figures 2(c) and 2(d)). Diercks et al. [19] also reported that the human N4BP2 C-terminal domain has a tertiary structure homologous to that of the N-terminal domain of bovine DNase I (2DNJ) (Z-score: 4.5). The primary structure comparisons between the *T. thermophilus* MutS2 Smr domain and the catalytic domain of *E. coli *RNase E or the N-terminal domain of bovine DNase I are shown in Figures 2(e) and 2(f), respectively. Although the HGXG/TGXG motif in loop 3 is not found in *E. coli *RNase E and bovine DNase I, some of the residues required for the catalytic activity of RNase E or for the DNA-binding activity of DNase I seem to be conserved in the Smr domain (Figures 2(e) and 2(f)). Interestingly, bovine DNase I has a C-terminal catalytic domain whose primary and tertiary structures are similar to those of its N-terminal domain [23]. It has been pointed out that the C-terminal catalytic domain of bovine DNase I also shows slight structural similarity to the Smr domain [19]. 

 In addition to *E. coli* RNase E and bovine DNase I, a variety of proteins reveal a structural similarity to the Smr domains. For example, a similarity between the human N4BP2 Smr domain and the following proteins was reported [19]: *E. coli* YhhP, a putative cell division protein with an RNA-binding activity (1DCJ) [24] (Z-score: 7.4), the C-terminal domain of *Bacillus stearothermophilus* IF3C, a translational initiation factor (1TIG) [25] (Z-score: 6.0), the N-terminal subdomain of the* B. stearothermophilus* ribosomal S8 protein (1SEI) [26] (Z-score: 5.9), and the R3H domain, a putative single-stranded nucleic acid-binding domain of human Smubp-2 (1MSZ) [27] (Z-score: 4.9). Thus, Smr domains share a widely conserved fold with various kinds of oligonucleotide-binding proteins. 

## 4. All 3 Subfamilies of Smr Domains Have Endonuclease Activity

As mentioned above, the hypothesis that the Smr domain has an endonuclease activity has been proposed on the basis of the domain-architecture analogy of MutS2 to another endonuclease domain-containing MutS homologue [17]. The first experimental evidence for the endonuclease activity of the Smr domain was provided by the functional characterization of the C-terminal domain of human N4BP2, a family 2 Smr domain. The recombinant C-terminal domain of human N4BP2 incised a supercoiled plasmid DNA to generate an open circular form of the plasmid, demonstrating the nicking endonuclease activity of this protein [19, 21]. Next, the endonuclease activity of family 1 Smr domain was also confirmed. The Smr domain of *T. thermophilus* MutS2 was shown to relax supercoiled plasmid DNA and digest linear double-stranded DNA [28, 29]. Finally, it was demonstrated that *E. coli *YdaL, a family 3 Smr domain, exhibits endonuclease activity against supercoiled plasmid DNA [20]. Thus, all of 3 subfamilies of Smr domain have been verified to be endonuclease domains.

 The *k*_cat_ and *K*_M_ values for the endonuclease activity of the family 1 Smr domain against linear double-stranded DNA have been reported to be 0.041 min^−1^ and 290 nM, respectively [18]. The *k*_cat_ value is at least 20 times higher than that for a DNA mismatch repair nicking endonuclease MutL [30]. However, it would be appropriate to avoid kinetic parameter-based discussions until the parameters are determined using the most favorable substrate for each Smr domain. 

In order to elucidate the mechanism by which the Smr domain incises DNA, the products generated by Smr reaction were analyzed by mass spectrometry and the chemical nature of the cleaved DNA termini was identified. The result clearly showed that products of the Smr reaction contain 3′-hydroxy and 5′-phosphate termini, indicating that Smr hydrolyzes the phosphodiester bond of the deoxyoligonucleotides at 5′-side of the phosphate [31]. It has been also shown that the Smr domains require divalent metal cations for the reaction [19, 20, 29]. Generally, divalent metal ion-dependent nucleases require acidic amino acid residues to coordinate the metal ions [32, 33]. However, the catalytic residues of the Smr domain have not yet been identified to date. 

The pH dependence of the *k*_cat_ value of the *T. thermophilus* MutS2 Smr domain suggested that the endonuclease activity depends on the basic form of the amino acid side chain, which has a p*K*_a_ value around 6 [18]. In *T. thermophilus *MutS2, substitution of His-701 with alanine resulted in a drastic decrease in the velocity of the activity [18]. His-701 of the* T. thermophilus *MutS2 is within the HGXG sequence motif that is conserved in the family 1 and 3 Smr domains (Figure 1(a)). Nevertheless, the histidine residue in the HGXG motif is not conserved in the family 2 Smr domain. In addition, residual activity of H701A mutant of *T. thermophilus *MutS2 was observed [29], implying the involvement of other amino acid residues in the catalysis. 

The structural homology of Smr domains to *E. coli* RNase E and bovine DNase I may provide the clue to explore the catalytic residues of Smr domains. Asp-303 and Asp-346 in *E. coli* RNase E are the catalytic residue coordinating a magnesium ion, and Asn-305 supports the orientation of Asp-303 via hydrogen bonding [22] (Figure 2(e)). The majority of Smr domains contain an aspartate at the site (Asp-669 and Asp-1692 in the *T. thermophilus* MutS2 and* H. sapiens* N4BP2 Smr domains, resp.) that spatially corresponds to Asp-303 of* E. coli* RNase E (Figures 1 and 2(e)). Furthermore, the orientation of Asp-669 in the *T. thermophilus* MutS2 Smr domain is adjusted by a salt bridge with Arg-671 [14], which is located in the site corresponding to Asn-305 of *E. coli* RNase E (Figure 2(g)). The involvement of those aspartate residues may be suspicious; however,* E. coli *YdaL lacks this aspartate residue. 

Primary structure comparison revealed that Glu-677 in* T. thermophilus* MutS2 shows relatively high level of conservation among all 3 Smr subfamilies [11], implying the possible involvement of this acidic residue as a catalytic residue. However, *E. coli *YdaL also does not have an acidic residue at the corresponding site. It would be possible that 3 subfamilies employ different amino acid residues to catalyze the reaction. 

The residues required for the DNA-binding ability of the family 2 Smr domain (human N4BP2 Smr domain) were surveyed in detail by using NMR measurement and site-directed mutagenesis [19]. The residues whose chemical shifts were affected by addition of bubble DNA structure are mapped to loops 1, 3, 4, and 5 of the human N4BP2 Smr domain. Subsequent site-directed mutagenesis confirmed the significant requirements of the basic and neutral residues in loops 2 (Lys-1722), 3 (Ser-1735, Arg-1741, and Lys-1743), and 4 (Arg-1756) for DNA-binding activity. Since the residues around loop 4 are poorly conserved, Diercks et al. [19] discussed the possibility that DNA-binding induced secondary effects on the local structures of these residues. While Arg-1741 and Lys-1743 are relatively conserved in all 3 Smr subfamilies, Lys-1722 seems to exist only in family 2, and Ser-1735 is conserved only in families 1 and 2 (Figure 1). It should be noted that Arg-1741 and Ser-1735 are located near the site spatially corresponding to the DNA-binding residues of bovine DNase I [19]. The DNA-binding mode of a single polypeptide of the Smr domain may be analogous to that of DNase I. However, as discussed later, we should take into account the quaternary structure of the Smr domains when we consider their DNA-binding mode. 

## 5. Substrate Specificity of Smr Domains

It has been reported that MutS2 preferably binds to branched DNA structures, such as Holliday junctions, D-loops, and pseudo-Y structures [10, 11, 14]. Its binding specificity for branched DNA structures is analogous to that of another MutS paralogue, MutS*γ* (that comprises MSH4 and MSH5) [34, 35], which does not contain Smr domain. Therefore, the involvement of the Smr domain in the recognition of branched DNA structures was unexpected. In fact, the binding affinity of the Smr-deleted mutant (N-terminal domain) of *T. thermophilus *MutS2 is as tight as that of the intact MutS2 [14]. However, surprisingly, the Smr domain of *T. thermophilus* MutS2 showed specificity to the Holliday junction though its *K*_d_ value (260 nM) was significantly higher than that of the N-terminal domain (60 nM) [14]. In addition, it was also reported that the human N4BP2 Smr domain and the *E. coli *YdaL domain showed a significant binding preference for branched DNA structures, including bubble DNA structure and Holliday junctions [14, 19]. Substrate specificity for branched DNA structures is a common feature among all 3 Smr subfamilies. 

Generally, branched DNA-recognizing proteins, with a few exceptions, are dimeric or tetrameric molecules, because they need to hold multiple “arms” of the substrate. For instance, T7 endonuclease I [36], T4 endonuclease VII [37], eukaryotic MutS*γ* (MSH4/MSH5) [34], eukaryotic MUS81-EME1 [38], and archaeal Hef [39] function in dimeric forms, and bacterial RuvA [40] and RuvC [41] are known to be tetrameric. Therefore, it is possible that Smr domains are in an oligomeric state in their functional form, although the three-dimensional structure of oligomerized Smr domains has not yet been reported. As to family 1 Smr domain, 621–662 residues of *T. thermophilus *MutS2, which are located between the N-terminal and the Smr domains, are responsible for the dimerization of the Smr domain [14, 18]. Consistent with this, a family 2 Smr domain, the human N4BP2 Smr domain, also forms a dimeric molecule upon DNA binding [19]. Recently, the DNA-binding and endonuclease activities of a family 3 Smr, *E. coli* YdaL, were found to be enhanced by the presence of the N-terminal 1–38 residues that are not included in Smr core domain [20]. Those N-terminal residues may affect the oligomeric state of* E. coli *YdaL. It has been known that *E. coli* RNase E also functions in a dimeric form [22] and that its dimer interface is located in the core region of the catalytic domain (the underlined region in Figure 2(b)), suggesting that the quaternary structures of the Smr domain and the *E. coli* RNase E catalytic domain are quite dissimilar to each other. The quaternary structure is likely to be closely correlated to the substrate specificity of these widely distributed folds of proteins.

## 6. Molecular and Cellular Functions of Smr-Containing Proteins

As mentioned above, a variety of Smr-containing proteins are distributed across a wide range of organisms. The biochemical characterization of Smr domains would improve the understanding of their cellular functions. 

 The family 1 Smr-containing protein MutS2 has been implicated to participate not only in the suppression of homologous recombination but also in the protection of cells from oxidative DNA damages [42, 43]. *Helicobacter pylori *MutS2 recognizes DNA containing 8-oxoguanine, a major DNA lesion caused by oxidative stress, and deletion of *mutS2* gene results in an accumulation of 8-oxoguanine in the cell [43]. Endonuclease activities are often required for DNA repair pathway to conduct the downstream excision reactions of damaged nucleotides [2]. It would be intriguing to test the activity of the Smr domain on 8-oxoguanine-containing DNA.

Family 2 Smr-containing proteins are extreme diverse in their domain architecture. Among these, the plant GUN1 and the mammalian N4BP2 are relatively well characterized. *Arabidopsis thaliana* GUN1 was identified as the key component in the plastid-to-nucleus retrograde signaling pathways that couple nuclear gene expressions and chloroplast functions [44]. GUN1, a member of pentatricopeptide repeat- (PPR-) containing proteins, has a Smr domain in its C-terminal region. Most of the PPR-containing proteins are thought to function in processing and stabilizing RNA molecules [45, 46], as well as in interacting with DNA molecules [47]. It was confirmed that the GUN1 Smr domain binds to DNA, and its binding activity was affected by the PPR motif [44]. Further experiments revealed that on plastid DNA, GUN1 is located at the sites that are being actively transcribed [44]. In addition to GUN1,* Arabidopsis thaliana* has at least 7 GUN1 paralogues that contain both the PPR motif and the Smr domain [48]. One of them, pTAC2, has been reported to colocalize with GUN1 at the site of actively transcribed plastid and thought to be responsible for the plastid gene expression [48, 49]. Another GUN1 paralogue, SVR7, has been discussed to be directly involved in chloroplast rRNA processing [48], where the endonuclease activity may be required. 

Mammalian N4BP2 was originally identified as a protein that specifically interacts with the E3 ubiquitin ligase NEDD4 [50]. Subsequent studies also revealed specific interaction of N4BP2 with BCL3 [21]. BCL3 is thought to activate transcription by interacting with transcription factors and other DNA-binding proteins [51, 52]. Induction of both NEDD4 and BCL3 is known to be correlated with various types of cancer including human breast cancer [53, 54], and the association of N4BP2 itself with sporadic carcinoma has also been reported [55]. It remains to be investigated whether the branched DNA-specific binding and/or the endonuclease activity of the Smr domain is involved in the transcription-regulatory role of N4BP2. It should be mentioned that a highly conserved domain of unknown function DUF1771 (in Pfam [56]) is often adjacent to family 2 Smr-domain in eukaryotes (Table 1). The Smr domains in mammalian N4BP2 are also accompanied by DUF1771. Structural and functional analyses of DUF1771 would provide information important for illustrating the molecular function of family 2 Smr domains. 

*Lactobacillus casei *phage *ϕ*FSW repressor can be classified as a family 3 Smr domain [16]. It would be worth elucidating whether the repressor protein has endonuclease activity. The *ϕ*FSW repressor protein has no N-terminal stretch, and its molecular and cellular function may be distinct from those of other family 3 Smr domains. To date, there are no reports about the cellular functions of other stand-alone type Smr domains. 

The transcription regulatory role of the *ϕ*FSW repressor protein is reminiscent of those of GUN1 and N4BP2. Although an endonuclease activity hardly seems to be correlated with the regulation of transcription, it has been shown that human NM23-H2 is a transcriptional regulator with DNA-cleaving activity [57, 58]. Furthermore, it has been clarified that mammalian nucleotide excision repair (NER) components including XPG and ERCC1-XPF endonucleases are recruited to the transcription machinery at the promoter of nuclear receptor genes [59]. NER is known to function in transcription-coupled repair, which rescues the stalled RNA polymerase II by repairing DNA lesions and requires CSB protein as a mediator [60, 61]. However, the recruitment of NER endonucleases to the promoters is independent of the exogenous genotoxic agents and transcription coupling repair-specific CSB [62]. Thus, in addition to transcription-coupled repair of DNA lesion, NER endonucleases may also participate in transcription itself. Le May et al. discussed the possible role of NER components in chromatin remodeling during the transcription [59, 62]. These observations lead us to the supposition that DNA-cleaving activity of Smr domain may play a role in the regulation of transcription. There is, of course, another possibility that *ϕ*FSW repressor protein, GUN1, and N4BP2 have multiple cellular functions and the endonuclease activity is not correlated with the regulation of transcription.

## 7. Conclusions

The sequences homologous to the Smr domains of MutS2 proteins are conserved in almost all organisms except for archaea. Smr domains are classified into 3 subfamilies on the basis of the domain architecture of the proteins in which Smr domains are present. Three-dimensional structures of Smr domains revealed that all 3 subfamilies share a common overall structure despite the local differences in loop regions. Consistent with this, all 3 subfamilies showed endonuclease activity and specificity for branched DNA structures. Immediate identification of the catalytic residues is required to study the reaction mechanism of this endonuclease. Since the relationship between the cellular and molecular functions of the majority of the family 2 or 3 Smr domain-containing proteins is still unknown, detailed characterization of these Smr domains may lead to the discovery of a novel biological phenomenon. For this purpose, an unavoidable task in the future will be to identify the most preferable substrate of the endonuclease or DNA-binding activity.

## Figures and Tables

**Table 1:** Distribution of Smr domain-containing proteins.

Species	Proteins containing family 1 Smr domains	Proteins containing family 2 Smr domains	Proteins containing family 3 Smr domains
*Thermus thermophilus*	MutS2 (YP_144911)^∗1^	—	—
*Helicobacter pylori*	MutS2 (ZP_03440043)	—	—
*Bacillus subtilis*	MutS2 (NP_390736)	—	—
*Deinococcus radiodurans*	MutS2 (NP_295699)	—	—
*Aquifex aeolicus*	MutS2 (NP_213851)	—	—
*Thermotoga maritima*	MutS2 (NP_229083)	—	—
*Arabidopsis thaliana*	MutS2 (NP_200220)	GUN1 (NP_849962)	—
		pTAC2 (NP_177623)	
		SVR7 (Q8GWE0.2)	
		At5G46580 (NP_199470)	
		At1G79490 (NP_178067)	
		At1G18900 (NP_973860)	
		At1G74750 (NP_177613)	
		At2G17033 (NP_849962)	
*Escherichia coli*	—	—	YdaL (NP_415856)^∗5^
			YfcN (AP_002931)
*Neisseria gonorrhoeae*	—	—	NGO0880 (YP_207992)
*Pseudomonas aeruginosa*	—	—	Hypothetical protein (AAG_05064)
*Saccharomyces cerevisiae*	—	CUE2 protein (EEU_05137)	—
		Ypl199cp (NP_015125)^∗2^	
*Caenorhabditis elegans*	—	Hypothetical protein (NP_498004)	Hypothetical protein (NP_494494)
			Hypothetical protein (NP_494390)
*Drosophila melanogaster*	—	CG7139, isoform A (NP_649378)^∗3^	—
*Mus musculus*	—	N4BP2 (NP_001020088)^∗4^	—
*Homo sapiens*	—	N4BP2 (NP_060647)	—

^∗1^Numbers in parenthesis indicate accession numbers.

^∗2^Ypl199cp also contains DUF1771.

^∗3^CG7139 shows amino acid sequence similarity to mammalian N4BP2-like proteins. The highly conserved domain, DUF1771, is adjacent to the Smr domain in CG7139.

^∗4^DUF1771 is adjacent to the Smr domains in mammalian N4BP2.

^∗5^Organisms that possess a family 3 Smr domain do not have a family 1 Smr domain [11].
